# Iliopsoas Cross-Sectional Area at the Psoas Valley is Associated with Surgically Treated Femoroacetabular Impingement Syndrome: A CT-based Case–Control Study

**DOI:** 10.1007/s00264-026-06844-w

**Published:** 2026-05-13

**Authors:** Enejd Veizi, Nurdan Çay, Ahmet Fırat, Ayşegül Fırat

**Affiliations:** 1 Department of Orthopedic Surgery, Aspetar Orthopaedic and Sports Medicine Hospital, Doha, Qatar; 2 Department of Anatomy, Hacettepe University, Ankara, Turkey; 3 Department of Radiology, Aspetar Orthopaedic and Sports Medicine Hospital, Doha, Qatar; 4 Department of Orthopedic Surgery, VM Medical Park Ankara Hospital, Ankara, Turkey; 5 Department of Anatomy, Hacettepe University, Ankara, Turkey

**Keywords:** Hip, Iliopsoas, Anatomy, Hip arthroscopy, Femoroacetabular impingement syndrome

## Abstract

**Introduction:**

The iliopsoas muscle passes immediately anterior to the hip joint and lies in close proximity to the acetabular labrum at the level of the psoas valley. This anatomical relationship suggests that local muscle morphology may be associated with symptomatic hip pathology. The present study investigated the association between iliopsoas cross-sectional area at the psoas valley, adjacent osseous morphology, and surgically treated symptomatic hip-pathologies.

**Methods:**

In this retrospective case–control study, 92 adult patients who underwent hip arthroscopy between 2019 and 2024 and had preoperative computed tomography (CT) imaging were compared with 50 age- and sex-matched controls without documented hip pain who had CT scans obtained for non-hip-related indications. Three-dimensional CT reconstructions were used to measure retroinguinal and psoas-valley morphometric parameters, including iliopsoas crosssectional area at the level of the psoas valley. Multivariable logistic regression adjusted for age and body mass index was performed for variables that differed between groups.

**Results:**

The groups did not differ significantly in age, sex, body mass index, or side. The lacuna musculorum ratio was higher in the surgical cohort than in controls (0.5 ± 0.1 vs. 0.4 ± 0.1; p < 0.001). Iliopsoas cross-sectional area at the psoas valley was smaller in the surgical cohort (12.5 ± 3.2 cm2 vs. 13.7 ± 2.8 cm2; p = 0.025). In multivariable analysis, a higher lacuna musculorum ratio (OR:1.094, 95% CI 1.026–1.167; p = 0.006) and a smaller iliopsoas cross-sectional area (OR:0.998, 95% CI 0.996–1.000; p = 0.039) remained associated with membership in the surgically treated cohort.

**Conclusion:**

Smaller iliopsoas cross-sectional area at the psoas valley was more often associated with surgically treated hip pathologies. These findings support a possible anatomical relationship between anterior hip soft-tissue morphology and symptomatic hip pathology, but they do not establish a protective causal effect of greater iliopsoas muscle bulk against labral injury.

## ***Introduction***

The hip joint plays a critical role in transferring body weight to the lower extremities and demonstrates an integrated harmony among osseous structures, joints, ligaments, muscles, and neurovascular elements (1, 2). As one of the most important dynamic stabilizers of the hip, the iliopsoas muscle group serves as the primary contributor to hip flexion (2, 3). Originating from the lumbar vertebrae and the iliac fossa, this muscle group inserts onto the lesser trochanter of the femur and is essential for functions such as walking, running, and postural balance (4, 5). However, as it courses anterior to the hip joint capsule, its close anatomical relationship with the acetabular labrum may also provide a substrate for potential pathological interaction (4).

Interest in the iliopsoas region has increased as appreciation has grown for extra-articular contributors to hip symptoms, including iliopsoas impingement and subspine morphology (3, 6, 7). At the anterior acetabular rim, the psoas valley represents a focal depression that accommodates the passage of the iliopsoas complex.(8, 9). Prior anatomical and arthroscopic studies suggest that this region lies close to the direct anterior labrum, where focal labral lesions may occur. However, the precise relationship between the local bulk of the overlying iliopsoas muscle and symptomatic hip pathology remains unclear.

Most prior studies have focused on tendon position, snapping phenomena, or acetabular morphology rather than the amount of iliopsoas muscle tissue present at the level of the psoas valley (4, 8). This study, therefore, aimed to investigate whether iliopsoas cross-sectional area at the psoas valley, together with related osseous parameters, is associated with membership in a surgically treated symptomatic hip-arthroscopy cohort. We hypothesized that morphometric differences in this region would be detectable between surgically treated symptomatic patients and controls without documented hip pain.

## Methods

### Study groups

Patients undergoing a hip arthroscopy procedure between February 2019 and May 2024 at our medical centre were eligible for inclusion in this retrospective case–control study. Inclusion criteria were age ≥ 18 years, presence of a preoperative CT scan, presence of preoperative clinical score data and detailed information regarding the intraoperative findings. Exclusion criteria were age < 18 years, a diagnosis of ipsilateral or contralateral hip osteonecrosis or osteoarthritis, an associated periacetabular osteotomy procedure, absence of CT scan imaging, lack of intraoperative data regarding the status of the labrum, and a history of prior hip surgery.

For comparison, an age- and sex-matched control group of 50 individuals without documented hip pain was selected through anonymized PACS (picture archiving and communications system) screening. Controls had CT scans obtained for non-hip-related indications (such as intra-abdominal pathologies, ovarian cysts, etc.) and were screened against available outpatient records. Because the control cohort did not undergo arthroscopy or magnetic resonance arthrography, occult asymptomatic labral pathology could not be excluded.

**Fig. 1:**
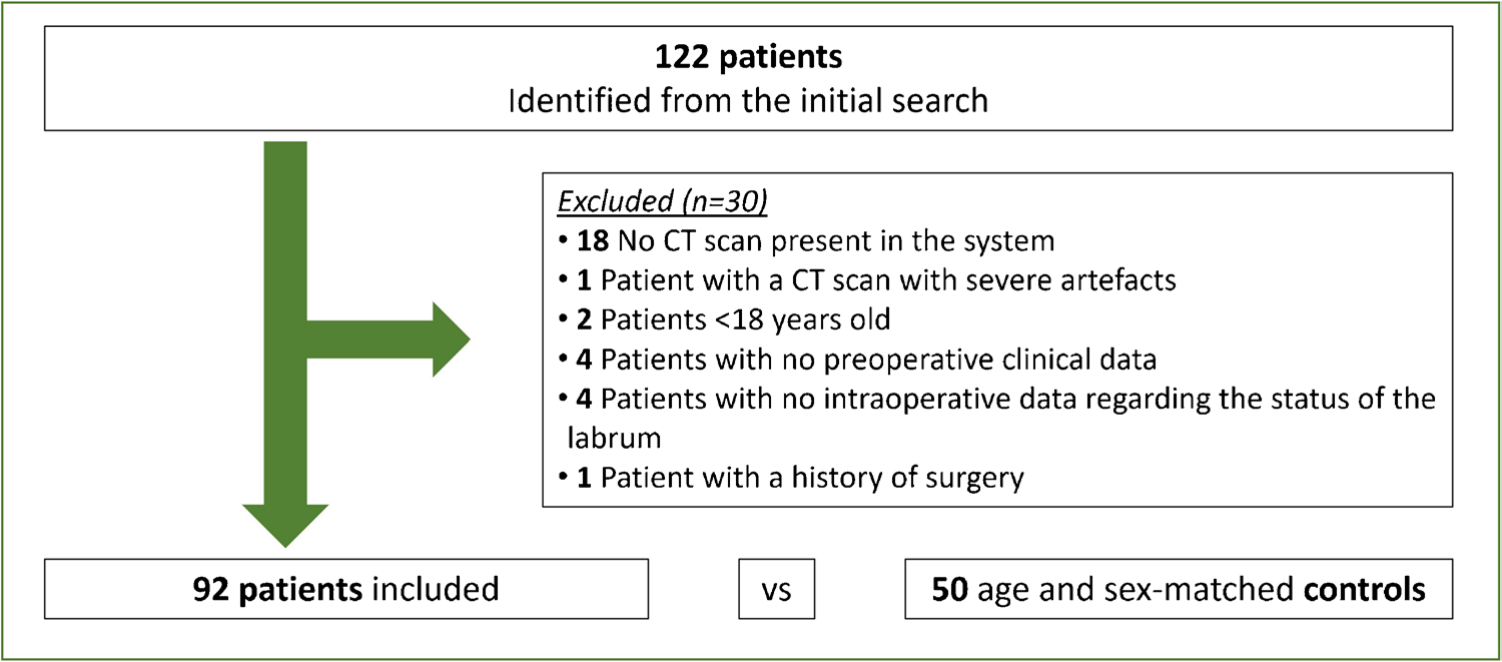
Study flowchart

Between the mentioned timeframe, 122 patients had undergone a hip arthroscopy procedure. Of them, 18 patients had no CT scan on the system, one patient had a CT scan influenced by the presence of metal artefacts, two patients were underage, one patient had a history of surgery on the ipsilateral hip, and eight patients lacked clinical data. The study group was comprised of the remaining 92 patients (Fig. 1).

Ethics approval was obtained from the Local Ethics Committee (date: 23.07.2024, nr. 2024/12—13) and all partecipants gave their written and verbal consent for their data to be used in scientific research. The study was conducted in line with the 1964 Declaration of Helsinki and its later amendments.

### Radiological and clinical evaluation

All radiological measurements were performed by an independent musculoskeletal radiologist who was blinded to surgical procedures and clinical findings. The measurements were performed twice and two weeks apart from each other, to assess for intra-observer reliability.

**Fig. 2:**
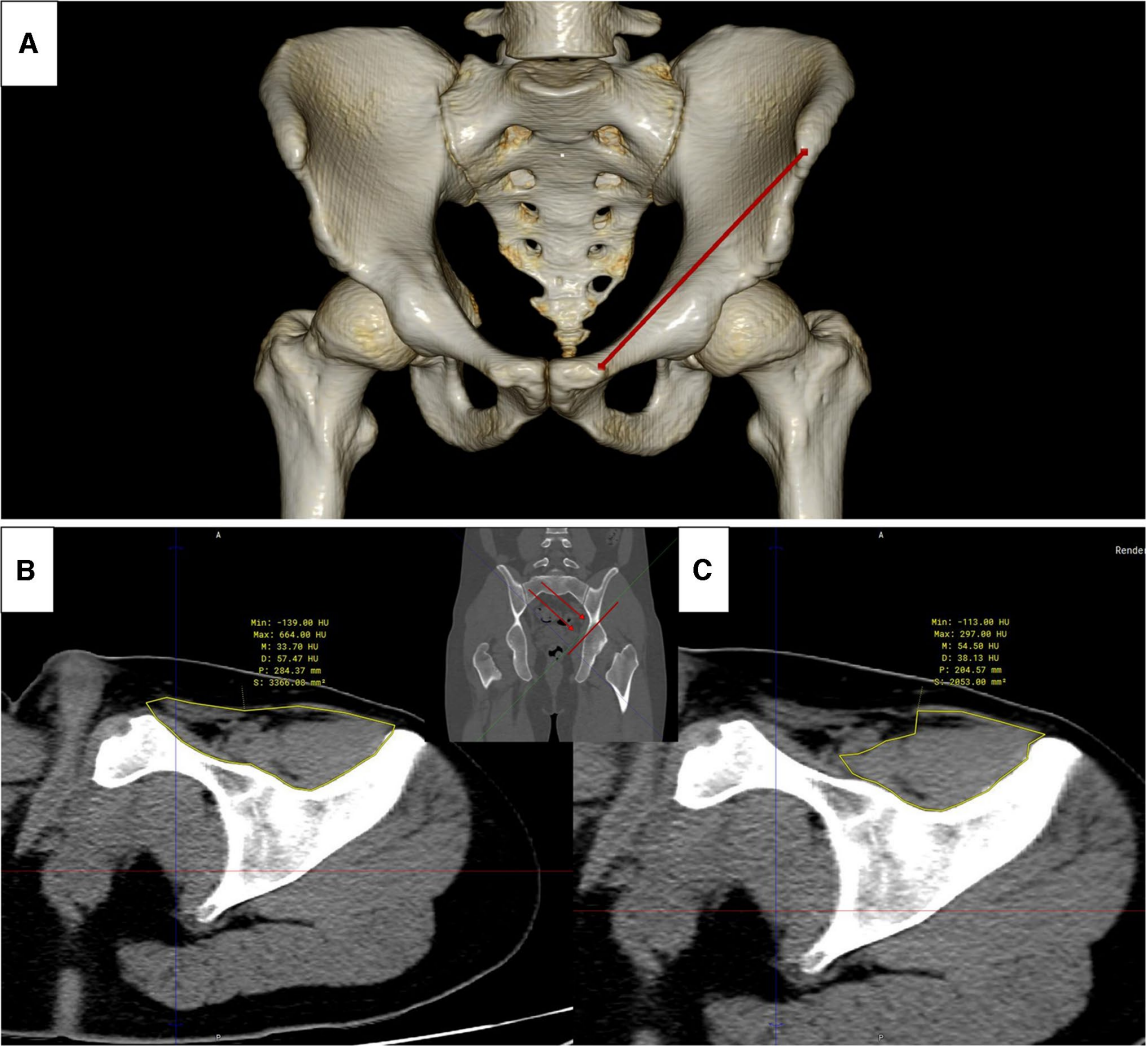
Inguinal ligament length measurement on 3D reconstructed CT scans (A). Using it as reference to align the planes, the cross-sectional area of the retroinguinal space (B) and of the lacuna musculorum (C) were measured on axial planes

The CT scans were non-contrasted and performed with a dual-source CT (Somatom® Definition Flash, Siemens Healthcare, Forchheim, Germany). Axial 1.5—mm slices were obtained and then multiplanar reconstruction was performed. Using 3-dimensional (3D) reconstructions of these CT scans from patients and controls, the following measurements were obtained:

**Inguinal ligament length**: measured between the anterior superior iliac spine and the pubic tubercle (Fig. 2A).

**Cross-sectional area of the retroinguinal space**: using the inguinal ligament as the reference line, the cross-sectional surface area of the retroinguinal space was measured (Fig. 2B).

**Lacuna musculorum area**: using the inguinal ligament as the reference, the surface area of the lacuna musculorum located lateral to the retroinguinal space was measured (Fig. 2C).

**Lacuna musculorum ratio**: defined as lacuna musculorum area/retroinguinal space area, representing the proportion of the retroinguinal space occupied by the lacuna musculorum.

**Fig. 3:**
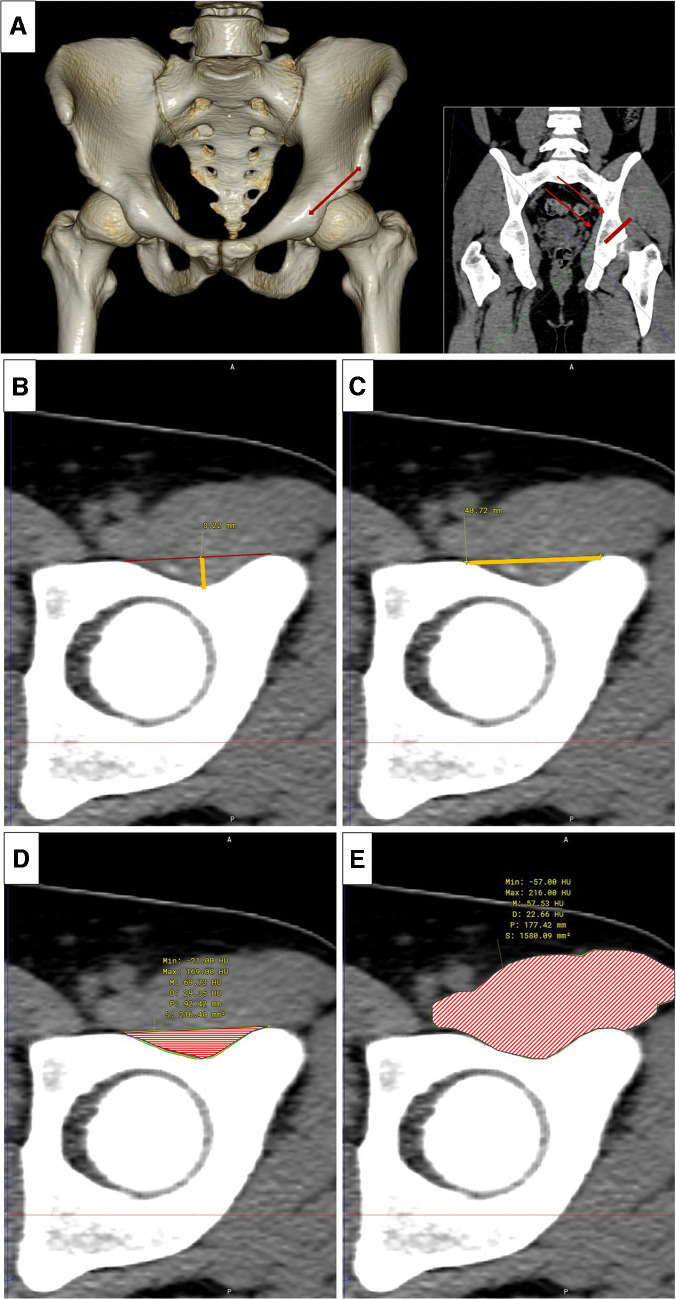
Identification of the borders of the psoas valley on 3D reconstructed CT scans (A). After aligning on the identified border, the valley’s depth (B) and width (C) were measured on the axial plane (orange lines). The cross-sectional area of the valley (D) and the muscle on top (E) were then measured on the same plane (red dashed area)

**Width and depth of the iliopsoas valley**: the valley was delineated on 3D CT images. The most prominent points on the 3D slices were selected as the start and end points. After defining its borders, width and depth were measured on axial CT slices (Fig. 3B, C).

**Psoas valley cross-section area**: the cross-section area enclosed within the borders defined above (Fig. 3D).

**Cross-sectional area of the iliopsoas muscle at the level of the valley**: on the axial slice corresponding to the psoas valley, the cross-sectional area of the overlying iliopsoas muscle was measured (Fig. 3E).

**Occupation ratio/overflow ratio**: calculated as iliopsoas muscle area/psoas valley area.

**Anterior inferior iliac spine (AIIS) type**: according to the classification by Hetsroni et al. (10), the AIIS is subdivided into three types:

Type 1: the most distal border of the AIIS lies proximal to the acetabular rim.

Type 2: the most distal border of the AIIS is contiguous with and ends at the same level as the acetabular rim.

Type 3: the most distal border of the AIIS extends beyond the acetabular rim and projects distally.

**Acetabular anteversion**: as previously described (10), a reference line was placed on sagittal images at the 1 o’clock and 11 o’clock positions of the acetabulum. Acetabular anteversion was then measured on the corresponding axial images using the Cobb function.

**Lateral centre–edge angle**: measured on coronal CT images and defined as the angle between the vertical plane and a line drawn from the center of a best-fit circle encompassing the femoral head to the lateral acetabular rim.

The preoperative clinical status of the patient group was assessed using the Harris Hip Score (HHS) which is part of our routine preoperative assessment in patients scheduled for a hip arthroscopy.

## Statistical analysis

Data were analyzed using SPSS version 31.0 (IBM Corp, Armonk, NY, USA). Categorical variables are presented as counts and percentages. Continuous variables are reported as mean, standard deviation, median, minimum, and maximum. The distribution of the data was assessed for normality, and comparisons were performed between the study groups accordingly. For comparisons of categorical variables between groups, Fisher’s exact test, the continuity-corrected chi-square test, and Pearson’s chi-square test were used. The Mann–Whitney U test was applied to compare non-normally distributed continuous variables, whereas the independent-samples t test was used for normally distributed continuous variables.

Bivariate correlation analyses were performed to evaluate potential associations between psoas valley measurements and other demographic and radiological parameters. Binary logistic regression was used to assess associations between morphometric variables and surgical-cohort membership. Given the retrospective case–control design, these analyses were not intended to establish causality or to determine whether any anatomical parameter independently indicated the need for surgery. The outcome variables should be interpreted as membership in a surgically treated hip-arthroscopy cohort rather than as a direct biological marker of symptomatic pathology or surgical necessity. Finally, receiver operating characteristic (ROC) analysis was performed to determine the presence of cut-off values. To assess for for intra-observer reliability an intraclass correlation coefficient was calculated and showed a value of 0.793, demonstrating good reliability of the study measurements. For all tests, a p value < 0.05 was considered statistically significant.

## Results

The mean age was 35.2 ± 8.6 years in the patient group and 35.2 ± 8.3 years in the control group (p = 0.886). No statistically significant differences were identified between the two cohorts with respect to age, sex, or the side of the evaluated hip. All demographic data are presented in Table 1.

**Table 1:** Demographic characteristics of the study cohorts

	**Patient group** **(n = 92)**	**Control group** **(n = 50)**	**p-value**
Age Mean ± SD Median (min – max)	35.2 ± 8.6 35 (18–50)	35.2 ± 8.3 35 (18–50)	0.886
Sex Men Women	50 (54.3%) 42 (45.7%)	30 (60.0%) 20 (40.0%)	0.517
BMI (kg/m^2^) Mean ± SD Median (min – max)	24.8 ± 3.3 24.2 (17.0–34.9)	23.9 ± 3.1 23.5 (16.7–31.7)	0.123
Side Left Right	38 (41.3%) 54 (58.7%)	20 (40.0%) 30 (60.0%)	0.880

**Fig. 4:**
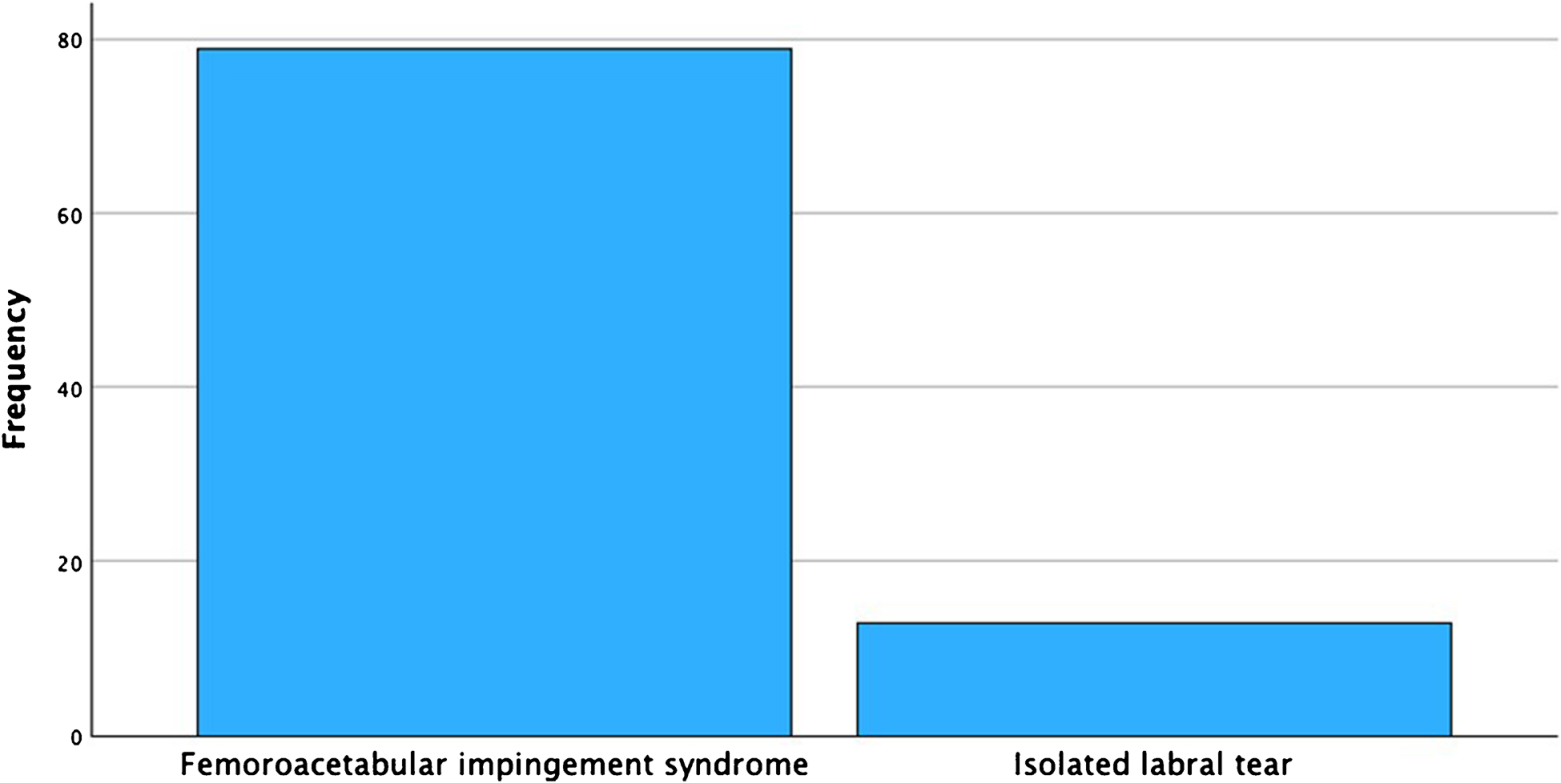
Diagnosis distribution of the patient group

The vast majority of patients underwent surgery with a diagnosis of femoroacetabular impingement (FAIS) (85.9%), whereas only 14.1% underwent hip arthroscopy for an isolated labral tear (Fig. 4).

Inguinal ligament length was similar between the patient and control groups (12.1 ± 0.9 cm vs 12.4 ± 0.9 cm, respectively; p = 0.388). Likewise, no significant between-group differences were observed in acetabular anteversion or the lateral center–edge angle (p = 0.675 and p = 0.480, respectively). The lacuna musculorum ratio was 0.5 ± 0.1 in the patient group and 0.4 ± 0.1 in the control group, with a statistically significant between-group difference (p < 0.001). Data related to radiological measurements are shown in Table 2.

**Table 2:** Morphometric measurements of the Lacuna Muscularis of the two study groups

	**Patient group** **(n = 92)**	**Control group** **(n = 50)**	**p-value**
Inguinal ligament’s length (cm) Mean ± SD Median (min – max)	12.1 ± 0.9 12.2 (9.4–14.7)	12.4 ± 0.9 12.3 (10.5–15.9)	0.388
Acetabular anteversion (degrees) Mean ± SD Median (min – max)	17.3 ± 8.0 17.4 (−14.4 – 36.2)	17.9 ± 5.6 18.4 (3.9–28.8)	0.675
Lateral Central Edge angle (degrees) Mean ± SD Median (min – max)	36.9 ± 8.6 37.0 (16.6–60.0)	36.0 ± 6.6 36.8 (17.7–54.6)	0.480
Retroinguinal space area (cm^2^) Mean ± SD Median (min – max)	29.0 ± 5.9 28.9 (17.5–45.7)	34.3 ± 8.5 34.8 (20.7–58.9)	** < *0.001***
Lacuna Muscularis area (cm^2^) Mean ± SD Median (min – max)	15,0 ± 4,5 14.9 (6.1–27.3)	14.9 ± 3.4 14.9 (9.0–22.0)	0.932
Lacunar occupational ratio Mean ± SD Median (min – max)	0.5 ± 0.1 0.5 (0.3–0.9)	0.4 ± 0.1 0.4 (0.3–0.6)	** < *0.001***

**Fig. 5:**
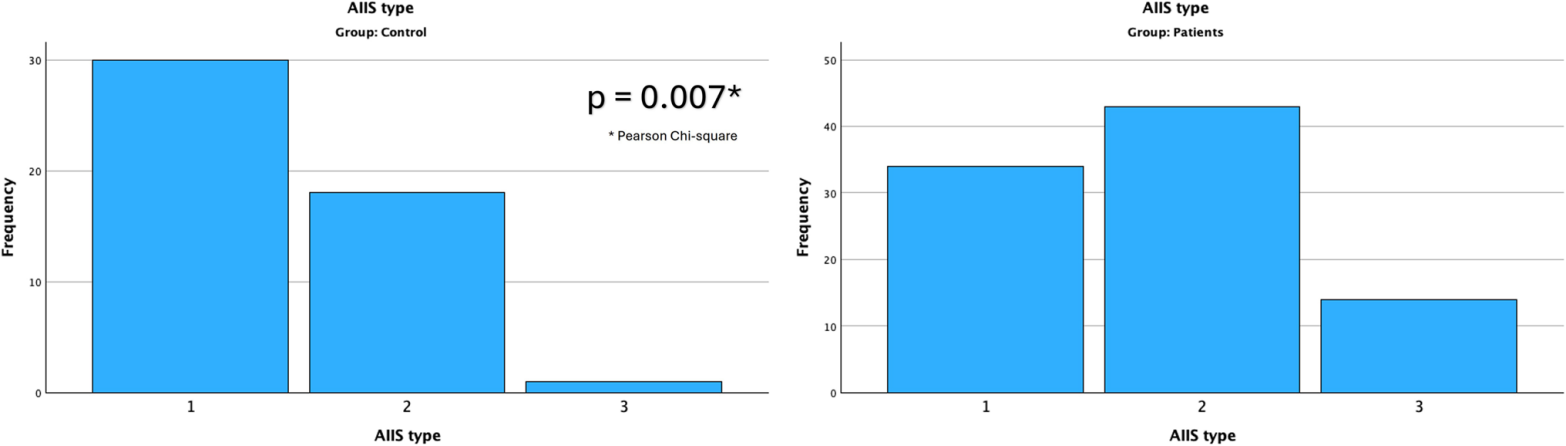
AIIS types according to study group

In the control group, the most common AIIS type was Type 1 (61.2%), whereas Type 2 was most frequent in the patient group (47.3%). Type 3 was identified in only one control patient (2.0%) but in 14 patients (15.4%) in the patient group. The distribution of AIIS types differed significantly between groups (p = 0.007) (Fig. 5).

While psoas valley depth was comparable between the patient and control groups, valley width was significantly greater in the control group (4.0 ± 0.4 cm vs 3.8 ± 0.6 cm; p = 0.018). The psoas valley cross-sectional area and occupation ratio were similar between groups (p = 0.598 and p = 0.165, respectively). In contrast, the cross-sectional area of the iliopsoas muscle overlying the psoas valley was significantly smaller in the patient group (12.5 ± 3.2 cm^2^ vs 13.7 ± 2.8 cm^2^; p = 0.025). Detailed data are provided in Table 3.

**Table 3:** Morphometric measurements and the occupational ratio of the Psoas Valley for the two study groups

	**Patient group** **(n = 92)**	**Control group** **(n = 50)**	**p-value**
Valley height (cm) Mean ± SD Median (min – max)	1.0 ± 0.3 1.0 (0.2–1.9)	1.0 ± 0.2 1.0 (0.6–1.5)	0.492
Valley length (cm) Mean ± SD Median (min – max)	3.8 ± 0.6 3.8 (1.6–5.0)	4.0 ± 0.4 4.0 (3.0–5.2)	***0.018***
Valley surface area (cm^2^) Mean ± SD Median (min – max)	2.5 ± 0.8 2.5 (0.2–4.5)	2.6 ± 0.6 2.6 (1.5–4.5)	0.598
Muscle area on top of psoas valley (cm^2^) Mean ± SD Median (min – max)	12.5 ± 3.2 12.9 (5.4–21.8)	13.7 ± 2.8 14.2 (8.3–19.0)	***0.025***
Occupational ratio of psoas valley Mean ± SD Median (min – max)	5.8 ± 4.4 5.0 (2.6–33.8)	5.5 ± 1.5 5.4 (2.8–9.5)	0.165

When stratified by sex, psoas valley width was similar in both sexes (3.9 ± 0.5 vs 3.9 ± 0.6 cm; p = 0.997), whereas depth was significantly greater in male patients (1.1 ± 0.2 vs 0.9 ± 0.2 cm; p = 0.002). Similarly, the cross-sectional area of the iliopsoas muscle overlying the psoas valley and the psoas valley occupation ratio were both significantly higher in males (p < 0.001 for both). Although acetabular anteversion was numerically higher in females, the difference did not reach statistical significance (p = 0.090). Finally, AIIS Type 2 was the most common type in males (n = 38, 48.1%), whereas AIIS Type 1 was most common in females (n = 34, 55.7%). All details are provided in Table 4.

**Table 4:** Morphometric measurements stratified by sex

	**Men** **(n = 80)**	**Women** **(n = 62)**	**p-value**
Valley depth (cm) Mean ± SD Median (min – max)	1.1 ± 0.2 1.1 (0.4–1.9)	0.9 ± 0.2 0.9 (0.2–1.5)	**0.002**
Valley width (cm) Mean ± SD Median (min – max)	3.9 ± 0.5 3.8 (2.6–4.9)	3.9 ± 0.6 3.8 (1.6–5.2)	0.997
Muscle area on top of psoas valley (cm^2^) Mean ± SD Median (min – max)	14.6 ± 2.4 14.9 (8.2–21.8)	10.8 ± 2.6 10.4 (5.4–18.1)	** < 0.001**
Occupational ratio of psoas valley Mean ± SD Median (min – max)	5.8 ± 1.8 5.6 (3.2–14.2)	5.6 ± 5.1 4.6 (2.6–33.8)	** < 0.001**
Acetabular anteversion (degree) Mean ± SD Median (min – max)	16.5 ± 7.9 16.8 (−14.4 – 33.1)	18.8 ± 6.1 18.3 (3.6–36.2)	0.090
AIIS type Type 1 Type 2 Type 3	30 (38.0%) 38 (48.1%) 11 (13.9%)	34 (55.7%) 23 (37.7%) 4 (6.6%)	0.083

Correlation analyses did not reveal significant associations between clinical scores or diagnostic subtypes and radiological variables. However, psoas valley depth and width demonstrated significant positive correlations with the cross-sectional area of the overlying muscle (r = 0.275, p < 0.001 and r = 0.215, p = 0.010, respectively). Details of the correlation analyses are presented in Tables 5 and 6.

**Table 5:** Correlation analysis of the morphometric measurements of the patient group and the preoperative clinical scores and diagnosis

Patients Group (n = 92)		Lacuna Muscularis area	Lacunar occupational ratio	Valley surface area	Muscle area on top of psoas valley	Occupational ratio of psoas valley
Clinical diagnosis (isolated labral tear vs FAIS)	Pearson correlation	−0.051	0.046	−0.052	−0.097	−0.053
	*p-value*	0.626	0.665	0.620	0.356	0.614
Preoperative HHS score	Pearson correlation	0.021	−0.074	−0.051	0.098	−0.059
	*p-value*	0.841	0.481	0.632	0.351	0.579

**Table 6:** Correlation analysis of the size (depth & length) of the psoas valley and morphometric measurements of the patient group

Patients Group (n = 92)		Muscle area on top of psoas valley	AIIS type	Acetabular anteversion
Valley depth (cm)	Pearson correlation	0.275	0.195	−0.112
	*p-value*	** < 0.001**	**0.021**	0.186
Valley width (cm)	Pearson correlation	0.215	0.017	0.128
	*p-value*	**0.010**	0.841	0.133

Univariate and multivariate regression analyses were performed for variables that showed significant between-group differences. The iliopsoas occupation ratio within the psoas valley was not associated with surgical requirement (OR 1.025; 95% CI 0.922–1.141; p = 0.644). In multivariate regression analysis, a higher lacuna musculorum ratio (OR 1.094; 95% CI 1.026–1.167; p = 0.006) and a smaller cross-sectional area of the iliopsoas muscle at the level of the psoas valley (OR 0.998; 95% CI 0.996–1.000; p = 0.039) were identified as independent risk factors for surgical requirement. All results are presented in Table 7.

**Table 7:** Univariate and multivariate regression analysis of the morphometrical measurements and the operational cohort (patients requiring surgery) adjusted for age and body mass index

	Univariate regression analysis		Multivariate regression analysis	
	Undergoing Surgery			
	OR (95% CI)	p value*	OR (95% CI)	p value*
Retroinguinal space area	0.999 (0.998—0.999)	** < 0.001**	0.999 (0.998—1.000)	0.082
Lacunar occupational ratio	1.057 (1.012—1.103)	**0.013**	1.094 (1.026—1.167)	**0.006**
Valley depth	0.953 (0.789—1.152)	0,619		
Valley width	0.935 (0.861—1.014)	0.106		
Muscle area on top of psoas valley	0.998 (0.997—1.000)	**0.025**	0.998 (0.996—1.000)	**0.039**
Occupational ratio of psoas valley	1.025 (0.922—1.141)	0.644		

**Fig. 6:**
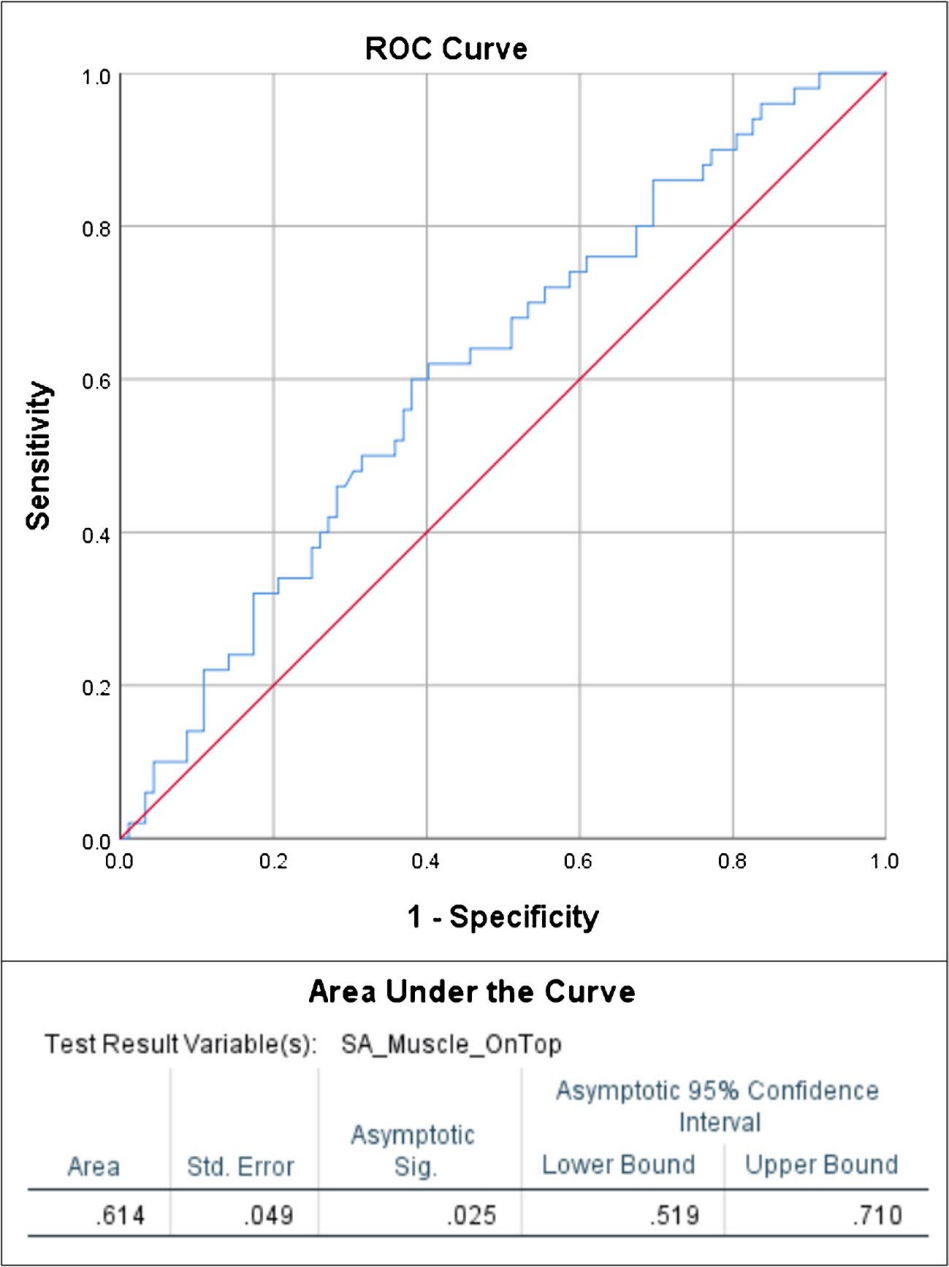
ROC analysis for the muscle cross-sectional area on top of the psoas valley

ROC analysis showed that the cross-sectional area of the iliopsoas muscle measured at the level of the psoas valley had a statistically significant, yet limited, discriminatory ability for the studied outcome (being in the control group/not requiring surgery). The area under the curve (AUC) was 0.614 (standard error 0.049), with a 95% confidence interval of 0.519–0.710. Iliopsoas muscle area performed better than chance in distinguishing between groups, however, the limited discriminatory capacity suggests that iliopsoas cross-sectional area should not be interpreted as a standalone clinical discriminator between patients requiring surgical treatment and controls (Fig. 6).

## Discussion

The principal finding of this study is that iliopsoas cross-sectional area measured at the level of the psoas valley was smaller in a surgically treated symptomatic hip-arthroscopy cohort than in controls without documented hip pain. The present findings should be interpreted as associations with a surgically treated cohort rather than evidence that iliopsoas morphology independently determines symptom development or surgical need. It is still unclear whether or not the occupation ratio of the iliopsoas within the valley or the overall muscle bulk immediately anterior to the labrum, provide any additional support against labral injury and degeneration. The absolute difference in iliopsoas cross-sectional area between groups was modest, and the ROC analysis demonstrated limited discriminatory performance. Therefore, the present findings should be regarded as hypothesis-generating anatomical observations rather than clinically actionable thresholds.

The mechanism of impingement around the hip joint is nowadays recognized as arising not only from intra-articular structures but also from extra-articular sources (7, 11, 12). Accordingly, interest in the iliopsoas muscle and the psoas valley has grown substantially in recent years, largely due to their close anatomical proximity to the hip joint. By evaluating these two anatomical entities together and comparing patients who required surgery with those who did not, this study contributes novel data to the existing body of knowledge.

Multiple authors have consistently reported that the psoas valley is located at approximately the 3 o’clock position on the acetabular clock-face reference of Mozingo et al. (13, 14). Philippon et al. defined this structure as the superior boundary of the anterior labral sulcus at the 3 o’clock position (13). From the standpoint of surgical anatomy, Ina et al. further detailed that the superior aspect of the psoas valley is typically located at 3:00, with its center at approximately 3:30, and emphasized that many labral lesions cluster in this region—an area where the labrum is relatively “smallest” and potentially more vulnerable (7). Despite the consistency of its location, the psoas valley demonstrates measurable morphological variability.

With respect to depth, Pellico et al.(15) and Sachdeva et al. (16) reported sex-stratified values in centimeters. In these two studies, depth values were 0.82 ± 0.16 cm and 1.26 ± 0.3 cm in males, and 0.80 ± 0.195 cm and 1.02 ± 0.18 cm in females, respectively. In our study, while psoas valley width was similar in both sexes (3.9 ± 0.5 vs 3.9 ± 0.6 cm), valley depth was significantly greater in male patients (1.1 ± 0.2 vs 0.9 ± 0.2 cm). Collectively, these findings support the notion that the psoas valley may be deeper in males.

Clinically, these anatomical relationships are important because the iliopsoas tendon and its variants course immediately anterior to the psoas valley region and are in close proximity to the anterior capsule and labrum. Lifshitz et al. defined the iliopsoas tendon as the tendon formed by the confluence of the psoas major and iliacus (3). They further noted that this tendon may be presented as single, double, or triple tendon bundles, and that the deeper portion of the tendon lies anterior and lateral to the labrum. Ina et al. reported that the iliopsoas tendon passes just anterior to the psoas valley (3:00–3:30 position) and functions as an anterior stabilizer at 0°–15° of hip flexion (7). From a pathological perspective, Kuroda et al. reported that, rather than classic femoroacetabular impingement syndrome, iliopsoas impingement may lead to focal labral tears in this characteristic region (8).

Beyond the broader definition of “iliopsoas syndrome”, arthroscopic series also support a distinct impingement phenotype characterized by a direct anterior acetabular labrum lesion at the 3 o’clock position (3). In their case series, Domb et al. described 36 hips with isolated direct anterior (3 o’clock) labral pathology in the absence of FAIS, bony anomalies, trauma, or other causes that could explain the labral tear (11). Clinically, all patients presented with anterior hip pain that increased with active flexion, and some reported a snapping sensation. On physical examination, focal tenderness over the iliopsoas, a positive impingement test, and pain/avoidance with resisted straight-leg raise were prominent findings (3, 11, 17). In this population, targeted injections have particular diagnostic value. While pain relief after intra-articular injection may be incomplete in some patients, many report more pronounced improvement following iliopsoas injection, supporting an extra-articular pain generator (18).

This study has several limitations. Due to its retrospective nature, the study is prone to type two error and bias. We tried to overcome this by including almost all of the patients operated in the defined time period. Second, the study focuses on patients operated with a hip arthroscopy and not specifically on iliopsoas impingement. Despite the frequent overlay of iliopsoas impingement with FAIS or labral tears, patients who did not receive surgical treatment and who were otherwise followed up conservatively might have been missed and therefore the result of the study might be limited in this aspect. Third, residual confounding factors should also be considered. Iliopsoas cross-sectional area may be influenced by sex, body habitus, total skeletal muscle mass, athletic participation, occupational loading, limb dominance, hip-flexor strength, duration of symptoms, pain-related disuse, and previous rehabilitation. Although age and BMI were included in the adjusted model, several of these variables were not available due to the retrospective design. Therefore, the observed association between iliopsoas area and surgical-cohort membership may partly reflect unmeasured differences in activity profile, functional demand, or global muscle mass rather than a direct local anatomical effect. Another important limitation concerns the control group. Although controls had no documented hip pain and underwent CT for non-hip-related indications, they did not undergo dedicated hip examination, magnetic resonance arthrography, or arthroscopy. Therefore, occult asymptomatic labral tears, cam or pincer morphology, or early impingement-related changes could not be excluded. Finally, the fact that we measured surface area instead of total volume could also be interpreted as a limitation of the study. Despite these drawbacks, the study has several strengths. It is the first time that the muscle characteristics surrounding the psoas valley are analyzed in correlation with surrounding bony landmarks, in a region where the joint capsule and the labrum are the weakest, providing opportunities for further clinical and anatomical research. The fact that the whole cohort was operated by a single surgeon over time provides standardization of surgical technique and standard diagnostic accuracy. And lastly, by identifying a measurable association between iliopsoas morphology and surgical-cohort membership, this study provides a basis for future prospective studies incorporating muscle strength testing, activity level, MRI-based muscle quality assessment, and longitudinal clinical outcomes.

## Conclusion

Smaller iliopsoas cross-sectional area at the psoas valley was more often associated with surgically treated hip pathologies. These findings support a possible anatomical relationship between anterior hip soft-tissue morphology and symptomatic hip pathology, but they do not establish a protective causal effect of greater iliopsoas muscle bulk against labral injury.

## Competing Interests

The authors declare no competing interests.

## Data Availability

No datasets were generated or analysed during the current study.
